# Using health-facility data to assess subnational coverage of maternal and child health indicators, Kenya

**DOI:** 10.2471/BLT.17.194399

**Published:** 2017-08-28

**Authors:** Isabella Maina, Pepela Wanjala, David Soti, Hillary Kipruto, Benson Droti, Ties Boerma

**Affiliations:** a Health Sector Monitoring and Evaluation Unit, Department of Policy Planning and Health Care finance, Ministry of Health, Nairobi, Kenya.; b Department of Preventive and Promotive Health, Ministry of Health, Nairobi, Kenya.; c Kenya Country Office, World Health Organization, Nairobi, Kenya.; d Regional Office for Africa, World Health Organization, Brazzaville, Congo.; e Center for Global Public Health, Department of Community Health Sciences, University of Manitoba, 750 Bannatyne Avenue, R3E0W2 Winnipeg, Manitoba, Canada.

## Abstract

**Objective:**

To develop a systematic approach to obtain the best possible national and subnational statistics for maternal and child health coverage indicators from routine health-facility data.

**Methods:**

Our approach aimed to obtain improved numerators and denominators for calculating coverage at the subnational level from health-facility data. This involved assessing data quality and determining adjustment factors for incomplete reporting by facilities, then estimating local target populations based on interventions with near-universal coverage (first antenatal visit and first dose of pentavalent vaccine). We applied the method to Kenya at the county level, where routine electronic reporting by facilities is in place via the district health information software system.

**Findings:**

Reporting completeness for facility data were well above 80% in all 47 counties and the consistency of data over time was good. Coverage of the first dose of pentavalent vaccine, adjusted for facility reporting completeness, was used to obtain estimates of the county target populations for maternal and child health indicators. The country and national statistics for the four-year period 2012/13 to 2015/16 showed good consistency with results of the 2014 Kenya demographic and health survey. Our results indicated a stagnation of immunization coverage in almost all counties, a rapid increase of facility-based deliveries and caesarean sections and limited progress in antenatal care coverage.

**Conclusion:**

While surveys will continue to be necessary to provide population-based data, web-based information systems for health facility reporting provide an opportunity for more frequent, local monitoring of progress, in maternal and child health.

## Résumé

**Objectif:**

Mettre au point une démarche systématique pour obtenir les meilleures statistiques nationales et infranationales possibles concernant les indicateurs de la couverture de santé maternelle et infantile à partir des données de routine des établissements de soins.

**Méthodes:**

Notre démarche visait à obtenir de meilleurs numérateurs et dénominateurs pour calculer la couverture à l'échelle infranationale à partir des données fournies par les établissements de soins. Il a fallu pour cela évaluer la qualité des données et définir des coefficients d'ajustement à appliquer aux rapports incomplets des établissements, puis estimer les populations locales ciblées en fonction des interventions relevant d'une couverture quasi-universelle (première visite prénatale et première dose de vaccin pentavalent). Nous avons appliqué cette méthode au Kenya, à l'échelle des comtés, où les établissements font des rapports électroniques de routine via le système d'information sanitaire des districts.

**Résultats:**

L'exhaustivité des données communiquées par les établissements était bien supérieure à 80% dans l'ensemble des 47 comtés et la cohérence des données au fil du temps était correcte. La couverture de la première dose de vaccin pentavalent, ajustée pour assurer l'exhaustivité des données communiquées par les établissements, a servi à obtenir une estimation des populations du comté ciblées par les indicateurs de santé maternelle et infantile. Les données agrégées des comtés pour la période 2012/2013 à 2015/2016 (quatre ans) étaient cohérentes avec les résultats de l'enquête démographique et sanitaire menée au Kenya en 2014. Nos résultats indiquaient une stagnation de la couverture vaccinale dans la quasi-totalité des comtés, une forte augmentation des accouchements en maternité et des césariennes ainsi qu'une progression limitée de la couverture de soins prénataux.

**Conclusion:**

S'il restera nécessaire d'effectuer des enquêtes pour disposer de données sur la population, les systèmes d'information en ligne sur lesquels les établissements communiquent leurs données permettent de faire un suivi local plus fréquent des progrès en matière de santé maternelle et infantile.

## Resumen

**Objetivo:**

Desarrollar un enfoque sistemático para obtener las mejores estadísticas posibles a nivel nacional y subnacional para indicadores de cobertura sanitaria de madres e hijos a partir de datos de centros sanitarios rutinarios.

**Métodos:**

Nuestro enfoque trataba de obtener numeradores y denominadores mejorados para calcular la cobertura a nivel subnacional a partir de datos de centros sanitarios. Esto implicaba una evaluación de la calidad de los datos y una determinación de los factores de ajuste para los informes incompletos de los centros y, posteriormente, una estimación de las poblaciones locales objetivo según las intervenciones con cobertura cuasiuniversal (primeras consultas prenatales y primera dosis de vacuna pentavalente). Se aplicó el método en Kenya a nivel nacional, donde los centros aplican los informes electrónicos rutinarios a través del sistema de software de información sanitaria por distritos.

**Resultados:**

La exhaustividad de los informes de los datos de los centros fue superior al 80% en los 47 países y la coherencia de los datos con el paso del tiempo resultó ser positiva. La cobertura de la primera dosis de la vacuna pentavalente, ajustada para completar los informes de los centros, se utilizó para obtener estimaciones de las poblaciones nacionales objetivo para los indicadores sanitarios de madres e hijos. El conjunto de datos de los países durante el periodo de cuatro años comprendido entre 2012/13 y 2015/16 mostró una buena coherencia con los resultados de la encuesta de demografía y salud realizada en Kenya en 2014. Nuestros resultados mostraron una paralización de la cobertura de inmunización en casi todos los países, un rápido aumento de los partos y cesáreas en centros y un avance limitado de la cobertura de atención prenatal.

**Conclusión:**

Si bien las encuestas seguirán siendo necesarias para ofrecer datos basados en la población, los sistemas de información basados en la web para los informes de centros sanitarios ofrecen la oportunidad de un control local más frecuente del progreso de la salud materna e infantil.

## ملخص

**الغرض:**

تقييم الأسلوب المنهجي للحصول على الإحصائيات الوطنية ودون الوطنية الممكنة لمؤشرات التغطية الصحية للأمهات والأطفال من واقع البيانات الروتينية الخاصة بالمنشآت الصحية.

**الطريقة:**

كان الأسلوب المنهجي الذي اتبعناه يهدف إلى الحصول على بسوط ومقامات محسّنة لاحتساب التغطية المقدمة على المستوى دون الوطني من واقع البيانات الخاصة بالمنشآت الصحية. وانطوى ذلك على تقييم الجودة النوعية للبيانات والوقوف على عوامل التعديل للتقارير غير المكتملة الواردة من المرافق الطبية، ثم تقييم الشرائح السكانية المحلية المستهدفة بناءً على عمليات التدخل مع التغطية شبه الشاملة (بما يشمل الزيارة الأولى خلال الحمل والجرعة الأولى للقاح الخماسي). وقمنا بتطبيق الطريقة على كينيا على مستوى الدولة، حيث يوجد بها نظام لرفع التقارير الإلكترونية الروتينية من جانب المرافق الطبية وذلك من خلال منظومة البرمجيات للمعلومات الصحية للقطاعات.

**النتائج:**

كان رفع تقارير الاكتمال لبيانات المرافق يتخطى نسبة 80‏% بدرجة كبيرة في جميع المقاطعات البالغ عددها 47 مقاطعة، وكان اتساق البيانات بمرور الوقت جيدًا. وتمت الاستعانة بتغطية الجرعة الأولى للقاح الخماسي – والتي تم تعديلها لضمان اكتمال التقارير حسب المرافق – للخروج بتقديرات عن القطاعات السكانية المستهدفة في المقاطعات فيما يتعلق بمؤشرات صحة الأمهات والأطفال. وقد أظهرت البيانات المجمعة من البلدان للفترة الممتدة لأربعة أعوام بدءًا من عام 12/2013 وحتى 15/2016 درجةً جيدة من الاتساق مع النتائج المستخلصة من المسح الديموغرافي والصحي الذي تم في كينيا عام 2014. وأشارت النتائج التي حصلنا عليها إلى حدوث جمود في مستوى تغطية التطعيم في جميع المقاطعات تقريبًا، فيما أشارت إلى حدوث زيادة سريعة في أعداد عمليات الولادة الطبيعية والقيصرية في المرافق الصحية، مع حدوث تقدم محدود في مستوى تغطية الرعاية خلال مرحلة الحمل.

**الاستنتاج:**

رغم أن الاستبيانات ستستمر في دورها كأداة ضرورية لتقديم البيانات المستندة إلى القطاعات السكانية، فإن نظم المعلومات المستندة إلى شبكة الإنترنت – والمخصصة لرفع التقارير من جانب المرافق الصحية – تقدم فرصة لمتابعة التقدم على المستوى المحلي بمعدل أكثر تواترًا فيما يتعلق بمجال صحة الأمهات والأطفال. ****

## 摘要

**目的：:**

制定系统方法以便根据医疗机构常规数据获取尽可能准确的国家和地方母婴健康覆盖率指标相关统计数据。

**方法：:**

我们的方法旨在获得经优化的分子和分母，以便根据医疗机构数据计算地方覆盖率。 包括在医疗机构所报告数据不完整的情况下，评估数据质量和确定调整系数，然后基于几乎全面覆盖的干预措施（第一次产前检查和五价疫苗的首针）估算当地目标人群。 我们将该方法应用于肯尼亚县级地区，从而可通过地区保健信息软件系统获取医疗机构提供的常规电子报告。

**结果:**

全部 47 个县的医疗机构数据报告完整度均高于 80％，而且长时间内数据的一致性较好。 使用根据医疗机构报告完整度而调整的五价疫苗首针覆盖率，以获取达到母婴健康指标的县级目标人群估计数量。 四年期间（2012/13 年度至 2015/16 年度）从各个县获取的汇总数据与 2014 年肯尼亚人口和健康调查的结果具有良好一致性。 我们的研究结果表明，几乎所有县级地区的疫苗覆盖率均处于停滞状态，医院分娩和剖腹产的人数迅速增加且产前保健覆盖率的增长极为有限。

**结论:**

尽管为了提供基于人群的数据仍然需要继续开展调查，但是基于网络的医疗机构报用信息系统为更加频繁地监控地方母婴健康进展情况提供了机会。

## Резюме

**Цель:**

Разработать систематический подход для получения наиболее точных национальных и субнациональных статистических данных по показателям охвата услугами систем здравоохранения для матерей и детей на основании регулярно сообщаемых данных по медико-санитарным учреждениям.

**Методы:**

Разработанный авторами подход был нацелен на получение более точных числителей и знаменателей для расчета охвата на субнациональном уровне на основании данных по медико-санитарным учреждениям. Он включал оценку качества данных и определение коэффициентов корректировки для неполной отчетности, полученной от медико-санитарных учреждений, а также определение численности местных целевых групп населения на основе мероприятий с практически всеобщим охватом (первое дородовое посещение и первая доза пятивалентной вакцины). Авторы применили этот метод в Кении на уровне округов, где электронная отчетность предоставляется медико-санитарными учреждениями с помощью окружной программной системы медико-санитарной информации.

**Результаты:**

Полнота отчетности для данных, полученных из медицинских учреждений, значительно превышала 80% во всех 47 округах, и согласованность данных с течением времени была хорошей. Данные об охвате первой дозой пятивалентной вакцины, скорректированные с учетом полноты отчетности медицинских учреждений, были использованы для определения численности целевых групп населения на окружном уровне по показателям здоровья матерей и младенцев. Совокупные данные из округов за четырехлетний период с 2012–2013 по 2015–2016 гг. по существу не противоречили результатам демографического и медико-санитарного обследования, проведенного в Кении в 2014 г. Полученные авторами результаты свидетельствуют о застое в отношении охвата иммунизацией почти во всех округах, о быстром росте количества естественных родов и кесаревых сечений в медицинских учреждениях и об ограниченном прогрессе в отношении охвата услугами дородового ухода.

**Вывод:**

**Обследования будут по-прежнему необходимы для получения данных на основе популяций. Благодаря сетевым информационным системам, используемым для представления информации медицинскими учреждениями, становится возможным более частый местный мониторинг прогресса в области охраны здоровья матери и ребенка*.*

## Introduction

Countries are increasingly focused on the assessment of performance of health programmes at the subnational level. The sustainable development goals further amplify the importance of local data to assess progress and allocate resources to reduce inequalities within countries.^1^ Coverage of maternal and child health interventions are among the most commonly used measures to monitor the implementation of health programmes at both national and subnational levels.

During the era of the millennium development goals, monitoring the progress of maternal and child health interventions relied heavily on national household surveys. These are conducted about once every five years and provide data on national-level trends and differentials in maternal and child health indicators.^2^

Health-facility data are another source of population-based statistics for selected maternal and child health and other indicators. For instance, immunization programmes use such data to obtain coverage estimates at the national and local levels.^3^ Many countries are using health-facility data to monitor annual progress and sometimes to conduct more advanced analyses.^4^^–^^7^ Scorecards ‒ for instance in the African Leaders Malaria Alliance initiative^8^ ‒ are increasingly popular and often based on local facility data.

In general, however, concerns about data quality have hampered the use of health-facility data to obtain population-based statistics. Incomplete and inaccurate reporting of events, and the challenge of estimating the size of the target populations, especially at subnational levels, may lead to implausible high (well over 100%) or low coverage results. Survey-based estimates of maternal and child health intervention coverage are considered reliable if the survey design and implementation are of high quality.^9^^–^^12^ These are often the preferred source to monitor trends and inequalities. 

While such surveys can also provide subnational data at the first administrative level (provinces, regions or counties), they do not meet the demand for local coverage data, both in terms of frequency (annual) and disaggregation down to the second administrative level (districts or subcounties).

Recent progress in the implementation of electronic web-based reporting systems allows for easier and faster reporting and better data quality control and feedback. The system most commonly used (in over 40 countries) is district health information software, version 2 (DHIS 2; Health Information Systems Programme, University of Oslo, Norway).^13^^,^^14^ Wider use of DHIS 2 could result in more accurate reporting on the numerators of the coverage indicators for child vaccinations or antenatal and delivery care. If target populations can be estimated more accurately, facility-based coverage can be used for monitoring trends at subnational levels.

The objective of this study was to develop a systematic approach to obtaining the best possible statistics for maternal and child health coverage indicators from health-facility data. The method focused on assessing and adjusting for incomplete reporting of event data from health facilities and on improving estimates of the target populations. We applied the method to Kenya using data from facilities in the 47 counties and from the Kenya demographic and health surveys.

## Methods

Table 1 summarizes the four steps of the method and its application in Kenya. The first step is to obtain data and statistics from different sources. The national bureau of statistics provides the official population projections, by age, sex and subnational unit. The most recent population-based survey provides statistics on the coverage of key interventions at national and subnational levels for a specified time period before the survey. Subnational levels include provinces, regions and counties but usually not districts. At the health facility level, data for key maternal and child health interventions – such as ANC first and fourth visit, place of delivery, Caesarean section, first and third dose of pentavalent vaccination and measles vaccination – are obtained for multiple years (preferably at least three years) to be able to assess consistency over time. In most countries using DHIS 2, these data are derived from paper-based recording and reporting in almost all facilities. The monthly facility reports are then sent to the district or subcounty health office where the data are entered into DHIS 2 and uploaded to the Internet. However, some facilities (mainly hospitals) enter the data directly into DHIS 2.

**Table 1:** Summary of the method for computing maternal and child health coverage statistics from health facility routine data, with an example from Kenya, 2016

Step	Method	Kenya, September 2016
Step 1. Obtain data from different sources	Obtain most recent household survey with national and subnational statistics. Identify indicators with universally high coverage	Data from Kenya demographic and health survey 2014. Coverage of first antenatal visit and first dose of pentavalent vaccine ≥ 95% in most counties (41 of 47)
	Use official population projections, by subnational unit and target age and sex groups	Projections for total population and population < 1 year old by county
	Obtain health facility reports on services provided and reporting rates	Four years of data by county for key maternal and child health indicators (2012/13 to 2015/16)
Step 2. Assess and adjust health facility reported data (numerators)	Assess completeness of facility reporting. Adjust for non-reporting by making assumptions about performance of non-reporting facilities, using an adjustment factor based on comparison with survey data	Good reporting rates during 2012/13 to 2015/16, but increasing over time, which may affect trends. Adjustment factor selected on the basis of comparison with Kenya demographic and health survey 2014 at county level
	Check consistency of coverage of interventions over time, by county, for key indicators: numbers of first antenatal visit and first dose of pentavalent vaccine; compare numbers of first and third doses of pentavalent vaccine	Good consistency over time for data on coverage of first antenatal visit and first dose of pentavalent vaccine. First pentavalent vaccination numbers slightly higher than first antenatal visit numbers, suggesting more complete reporting
Step 3. Compute target populations based on health-facility data (denominators)	Compute coverages of first antenatal visit and first dose of pentavalent vaccine with census projection-based denominators to assess coverage level and identify outliers	National coverage was 90–95% (2012/13 to 2015/16), but six northern counties had consistently > 120% coverage, 12 counties had unlikely low coverage (< 80%)
	Revise the target population for infants based on reported first antenatal visit or first dose of pentavalent vaccine numbers.	First dose of pentavalent vaccine numbers from facilities used as target population, adding 3.0% for non-coverage of first dose of pentavalent vaccination
	Derive target populations for pregnancies, deliveries and infants	Kenya demographic and health survey 2014 data used to estimate target populations
Step 4. Calculate coverages using adjusted numerators and improved denominators	Calculate indicators for antenatal care, immunizations, delivery and other services. Check national and county rates	National level for 2012–14 close to Kenya demographic and health survey 2014; good consistency at county level

Step 2 starts with assessing the quality of the numerator of the coverage indicator by analysing completeness of reporting and consistency over time. High levels of reporting (over 80% of health facilities reporting a specific indicator) are essential to be able to compute coverage rates. Internal consistency is checked in terms of trends over time for coverage of each indicator, as well as between first antenatal visit and first pentavalent vaccination, and between first and third pentavalent vaccinations, as recommended by the World Health Organization.^15^ Outliers, defined as more than two standard deviations from the mean values of the numerators for the multi-year period, are identified and corrected if no satisfactory explanation is found for the outlier value.

For the coverage calculations, we need to adjust for incomplete reporting by facilities. This involves making assumptions about the number of service outputs (pregnancy care, vaccinations, etc.) provided at facilities which did not report compared with those that reported. The adjustment can be expressed as follows:

*n*(adjusted) = *n + n(*1/(*c*)‒1)*k (1)

where *n*  is the number of service outputs, *c* is the reporting completeness, *k* is the adjustment factor. If we consider the missing reports an indication that no services were provided during the reporting period, then *k* = 0, and no adjustment is made for incomplete reporting. However, if facilities provided services but not at the same level as before reporting periods, the apparent incomplete reporting is an indication of a lower level of service provision; *k* in this case is between 0 and 1. In other cases, it may be assumed that services were provided at the same rate in non-reporting facilities as in reporting facilities, and so *k* = 1. Important considerations in the selection of a value of *k* are the extent to which large health facilities and private health facilities are reporting and engaged in the provision of the specific services. This is likely to be different for different services, resulting in different adjustment factors. Subsequently, the selection of the most likely value of *k* is done through a comparison of facility reports with the survey results, by selecting a value of *k* that brings the adjusted health facility statistic close to the survey statistic for a particular year with data from both sources.

Step 3 is about finding the best possible denominator or target population size. This is usually obtained from census projections by the country’s national bureau of statistics. Often, problems with the projected subnational denominators lead to unexpectedly high or low coverage rates. An alternative approach is to derive the population size from health facility data on indicators with near-universal coverage (at least 90%), such as the first antenatal visit or the first dose of pentavalent vaccine (normally given at 6 weeks of age). If the health facility reports are of good quality, and almost all children are vaccinated, the first vaccination or first antenatal visit numbers should be very close to the actual target populations. Only a small proportion is added to the reported first pentavalent vaccination or first antenatal visit numbers to account for those who did not receive them (< 10% of people, according to household surveys in many countries).^16^^,^^17^ The estimated young infant target population can then be used to obtain target populations for other maternal and child health coverage indicators (e.g. live births, deliveries, pregnancies and older infants), based on available statistics from recent surveys or other sources.

In step 4, the adjusted numbers and denominators are used to calculate the subnational coverages of immunizations, antenatal care (first and fourth visits) and facility-based deliveries. 

In the second half of 2016, we used data from Kenya to apply and refine the method. Health-facility data were analysed across the 47 counties for the fiscal years (1 July to 30 June) 2012/13, 2013/14, 2014/15 and 2015/16 and compared with survey results from the most recent Kenya demographic and health survey in 2014.^18^ Population projections were obtained from the 2009 Kenya national census.^19^

All calculations were done using Microsoft Office 365 *Excel* software version 1705 (Microsoft Corporation, Redmond, United States of America). The spreadsheet with data by county and the adjustment procedure are available from the corresponding author.

## Results

### Data quality assessment

In Kenya in 2015/16, the national reporting completeness for the vaccination reporting forms was high (93.7%; 69 470/83 179 expected monthly reports; Table 2) and <  80% in only one county. This represented a modest increase in reporting rates since 2012/13 (national rate 89.4%; 60 450/72 384; <  80% in 12 counties). Also, the reporting rates were high throughout the period for antenatal and delivery care forms (from 85.9%; 72 384/84 276, in 2012/13 to 94.9%; 83 179/87 684, monthly reports in 2015/16). There were no outliers at the level of the 47 counties, which indicates good consistency over time. The internal consistency between numbers of first and third doses of pentavalent vaccine was also good, since the first vaccination values were higher in all counties and years, as expected, and the size of the difference between the two doses corresponded well with the survey drop-out rates (Table 2). The numbers of first pentavalent vaccinations and first antenatal visits in counties were similar, as expected on the basis of the Kenya demographic and health survey 2014 results.^18^ The numbers of first pentavalent vaccinations exceeded first antenatal visits in most counties, suggesting more complete reporting. Therefore, we used first pentavalent vaccination as the key indicator to obtain denominators.

**Table 2:** Assessment of numerators and denominators and adjustments for coverage of the first dose of pentavalent vaccine from health-facility data, Kenya, 2012/13 to 2015/16

Variable	Year			
	2012/13	2013/14	2014/15	2015/16
**Numerator**				
Reported no. of vaccinations	1 204 657	1 226 621	1 253 995	1 270 117
Reporting completeness, %^a^	84.4	85.3	91.7	93.7
Adjusted no.^b^	1 260 167	1 279 415	1 282 366	1 291 389
**Denominator, census**				
Census projection, infants	1 316 843	1 356 076	1 397 189	1 439 845
Coverage, based on census projection, %	95.7	94.3	91.8	89.7
Coverage, from Kenya demographic and health survey 2014, %^c^	97.0	97.0	N/A	N/A
**Denominator, first dose of pentavalent vaccination**				
No. of infants, adjusted for non-vaccinated (3%)	1 299 141	1 318 985	1 322 027	1 331 329
Coverage of first dose of pentavalent vaccine, %^d^	97.0	97.0	97.0	97.0

N/A: not applicable.

^a^ Reporting rate is the number of reports received divided by the number of reports expected.

^b^ Using adjustment factor for incomplete reporting at facilities, *k* = 0.25.

^c^ Coverage by 12 months among children aged 12–23 months (i.e. in 2012/13, if the survey is on average mid-2014).

^d^ Total number of vaccinations reported (adjusted) / number of infants eligible for vaccination (adjusted for non-vaccinated) × 100.

Notes: Data were from routine reporting of health facilities via DHIS 2 (district health information software, version 2.0). Health facility data are for fiscal years (1 July to 30 June).

We selected the adjustment factors based on our knowledge of the maternal and child health programmes, the types of facilities where care was provided and comparison with the survey-based statistics. For vaccination coverage the adjustment factor *k* is likely to be low as vaccine supplies are directly linked to reporting. We used *k* = 0.25 as some vaccinations may still be given in non-reporting facilities. This value of *k* also had good agreement with the national coverage rate for first pentavalent vaccination in the demographic and health survey.^18^ For antenatal and especially delivery care, the non-reporting facilities included a higher proportion of private facilities and most of those provided pregnancy related-services. In the demographic and health survey, one-quarter of all facility-based deliveries took place in private health facilities. To adjust for incomplete reporting, *k* was set at 0.5 for antenatal care and at 1 for deliveries, bringing the health-facility-based rates close to coverage rates for the three years preceding the survey.

County coverage rates based on the census population projections indicated that there were major denominator issues. Several counties had denominators that were too low (six counties – all in the northern parts of Kenya – consistently had coverage estimates exceeding 120%), while other counties had unlikely low coverage of the first pentavalent vaccination (11 counties were consistently below 80%). Because of these challenges with the accuracy of the census projections at the county level, our confidence in the quality of the facility reports on first pentavalent vaccination and the near-universal national coverage of this vaccination in the household survey data, we used the numbers of children with first pentavalent vaccination as an alternative estimate of the denominator or target population at the county level. To obtain the target population size, we added an estimate of the number of non-immunized infants (3% of the target population, based on the demographic and health survey)^18^ to the adjusted number of vaccinations, resulting in 1.33 million infants eligible for vaccination in 2015/16. We used this denominator as target population for all vaccinations. For live births, we added 2.0% to the denominator to include neonatal deaths. For deliveries, we reduced the number of live births by 1.5% to allow for multiple births^20^ and added 2.0% to allow for stillbirths. For pregnancies, we added 3.0% to deliveries to account for fetal loss before stillbirths. Early fetal losses are generally not included as in Kenya these mostly occur before the first antenatal care visit is made (median first visit is made at 5.4 months according to the demographic and health survey).^18^

### Infant vaccination coverage

The four-year trend in vaccination coverage from facility data for all Kenya showed flat or slightly declining coverage for the third dose of pentavalent vaccine and for measles vaccine (Table 3). Third pentavalent vaccination levels were consistent with the survey-based statistics. Measles vaccination coverage was somewhat higher in the facility data than in the survey data (vaccinated by 12 months among children aged 12‒23 months), which may be due to facility reporting of some vaccinations given to children after their first birthday, as the values were very close to the values in the 2014 demographic and health survey, based on children aged 12–23 months. The full vaccination coverages were considerably higher in the facility data than in the survey data, and also implausibly high compared with the coverage of the specific vaccinations. It is likely that over-reporting of full vaccination status occurred in the facility reports.

**Table 3:** Coverage of infant vaccinations and maternity care from health facility and survey data, Kenya, 2012/13 to 2015/16

Indicators	Facility data^a^					Survey data			
	Year 2012/13	Year 2013/14	Year 2014/15	Year 2015/16		DHS 2014^b^		DHS 2012–2014^c^	MIS 2013–2015^d^
						Vaccinated among children 12–23 months	Vaccinated by 12 months		
**Infant vaccinations**									
No. of infants eligible for vaccination	1 299 141	1 318 985	1 322 027	1 331 329		3 777	3 777	N/A	N/A
First dose of pentavalent vaccine									
No. of infants vaccinated	1 260 167	1 279 415	1 282 366	1 291 389		3 683	3 664	N/A	N/A
Coverage, %	97.0	97.0	97.0	97.0		97.5	97.0		
Third dose of pentavalent vaccine									
No. of infants vaccinated	1 165 483	1 185 887	1 197 074	1 196 086		3 396	3 335	N/A	N/A
Coverage, %	89.7	89.9	90.5	89.8		89.9	88.3		
Measles vaccine									
No. of infants vaccinated	1 159 811	1 121 647	1 171 606	1 157 572		3 290	2 980	N/A	N/A
Coverage, %	89.3	85.0	88.6	86.9		87.1	78.9		
Full immunization coverage									
No. of infants vaccinated	1 081 394	1 041 468	1 094 094	1 104 023		2 829	2 693	N/A	N/A
Coverage, %	83.2	79.0	82.8	82.9		74.9	71.3		
**Maternity care**									
No. of women giving birth	1 331 750	1 352 091	1 355 210	1 364 745		N/A	N/A	10 378	1 776
No. of pregnant women	1 371 702	1 392 654	1 395 866	1 405 688		N/A	N/A	N/A	N/A
Antenatal visit: first									
No. of pregnant women attending	1 265 594	1 336 775	1 359 273	1 400 956		N/A	N/A	9 890	1 669
Coverage, %	92.3	96.0	97.4	99.7				95.3	94.0
Antenatal visits: four or more									
No. of pregnant women attending	543 936	604 384	702 575	723 897		N/A	N/A	5 791	1 092
Coverage, %	39.7	43.4	50.3	51.5				55.8	61.5
Delivery in health-care facility									
No. of health facility deliveries	815 959	956 097	998 896	1 049 285		N/A	N/A	6 642^e^	N/A
Coverage, %	61.3	70.7	73.7	76.9				64.0^e^	
Caesarean section delivery									
No. of caesarean section deliveries	103 785	121 789	134 892	147 463		N/A	N/A	903	N/A
Coverage, %	7.8	9.0	10.0	10.8				8.7	

DHS: Kenya demographic and health survey; MIS: Kenya malaria indicator survey; N/A: not applicable.

^a^ From routine reporting via DHIS 2 (district health information software, version 2.0), using adjusted numerators and denominators. Numerators for facility data were adjusted for incomplete reporting; denominators were derived from facility data on the first dose of pentavalent immunization.

^b^ From the Kenya demographic and health survey 2014.^18^

^c^ From the Kenya demographic and health survey 2014, based on recall of the survey respondents.^18^

^d^ From the Kenya malaria indicator survey 2015, based on recall of the survey respondents.^21^

^e^ Survey data are for numbers of births; facility data are for numbers of women delivering.

Notes: Health facility data are for fiscal years (1 July to 30 June).

**Fig. 1:**
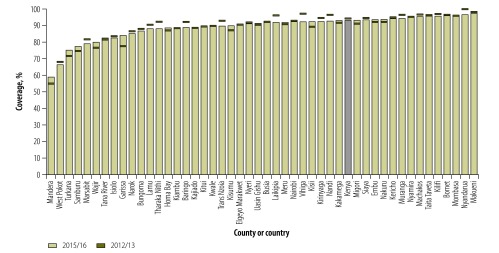
**Pentavalent vaccine coverage (receiving three doses in infancy) from health-facility data, by county, Kenya, 2012/13 and 2015/16**

Notes: Health-facility data were from routine reporting via DHIS 2 (district health information software, version 2.0) for fiscal years (1 July to 30 June). Adjusted values are shown, aggregated by county. Numerators for facility data were adjusted for incomplete reporting; denominators were derived from facility data on the first dose of pentavalent immunization. Coverage denominators for facility data are in Table 4. Kenya value, shown in grey, is the weighted mean of the 47 counties.

The differences by county within Kenya were substantial (Fig. 1). In 2015/16, 28 counties had third pentavalent vaccination coverage of 90% or higher, while five counties, all in northern Kenya, had third pentavalent vaccination coverage below 80%. In 25 of the 47 counties the third pentavalent vaccination coverages in 2015/16 were lower than in 2012/13.

**Table 4:** Numerators and denominators for calculating coverage of infant vaccinations (receiving three pentavalent doses in infancy) from health-facility data, by county, Kenya, 2012/13 and 2015/16

County or country	Year 2012/13		Year 2015/16	
	No. of eligible infants	No. vaccinated	No. of eligible infants	No. vaccinated
Baringo	19 314	17 743	18 747	16 676
Bomet	23 410	22 581	23 698	22 849
Bungoma	59 967	52 666	56 095	48 669
Busia	30 596	28 178	26 441	24 252
Elgeyo Marakwet	14 866	13 442	14 042	12 807
Embu	12 844	11 791	12 935	12 134
Garissa	16 458	12 734	18 846	15 869
Homa Bay	37 104	32 295	35 306	31 239
Isiolo	5 983	4 980	6 382	5 283
Kajiado	29 483	26 146	32 373	28 924
Kakamega	62 054	56 777	58 112	54 226
Kericho	24 414	22 958	22 746	21 231
Kiambu	45 260	42 913	54 254	51 177
Kilifi	45 271	39 908	46 596	41 433
Kirinyaga	11 098	10 729	11 635	11 160
Kisii	37 493	35 386	35 765	33 113
Kisumu	36 008	31 967	34 162	31 620
Kitui	29 326	25 518	27 203	24 493
Kwale	29 008	25 882	29 055	26 017
Laikipia	13 538	12 976	14 402	13 257
Lamu	4 177	3 766	4 417	3 902
Machakos	28 499	27 437	28 727	27 506
Makueni	22 646	22 220	20 273	19 772
Mandera	20 722	11 329	25 901	15 297
Marsabit	12 523	10 193	13 057	10 343
Meru	34 597	31 362	35 577	32 834
Migori	41 137	37 496	41 659	38 892
Mombasa	29 206	27 894	32 921	31 761
Muranga	21 577	20 852	20 296	19 182
Nairobi	118 297	109 680	133 044	123 088
Nakuru	57 852	53 217	61 295	57 653
Nandi	22 768	21 939	20 776	19 291
Narok	37 573	32 523	41 217	35 179
Nyamira	18 965	18 030	19 971	19 037
Nyandarua	14 871	14 860	15 082	14 606
Nyeri	16 290	14 885	15 126	13 802
Samburu	10 336	7 689	10 185	7 902
Siaya	31 409	29 592	27 275	25 561
Taita Taveta	7 347	7 060	7 858	7 531
Tana River	8 462	6 890	9 598	7 941
Tharaka Nithi	9 612	8 864	9 139	8 077
Trans Nzoia	26 374	24 468	28 015	25 183
Turkana	27 651	19 713	33 428	25 220
Uasin Gishu	30 160	27 255	32 664	29 872
Vihiga	18 106	17 508	18 233	16 874
Wajir	17 309	13 225	19 367	15 557
West Pokot	27 179	18 422	27 433	18 266
Kenya^a^	1 299 141	1 165 483	1 331 329	1 196 086

Notes: Health facility data are for fiscal years (1 July to 30 June).

^a^ Weighted county mean.

### Antenatal and delivery care

**Fig. 2:**
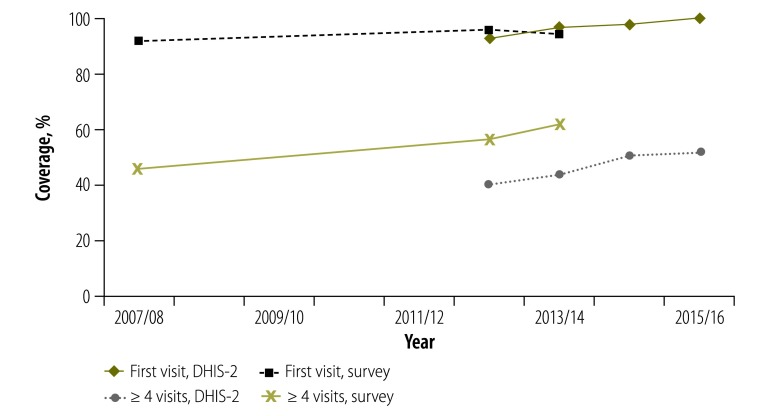
**Antenatal care visits coverage (first visit and four or more visits) comparing health facility and survey data, Kenya, 2007/08 to 2015/16**

**Fig. 3:**
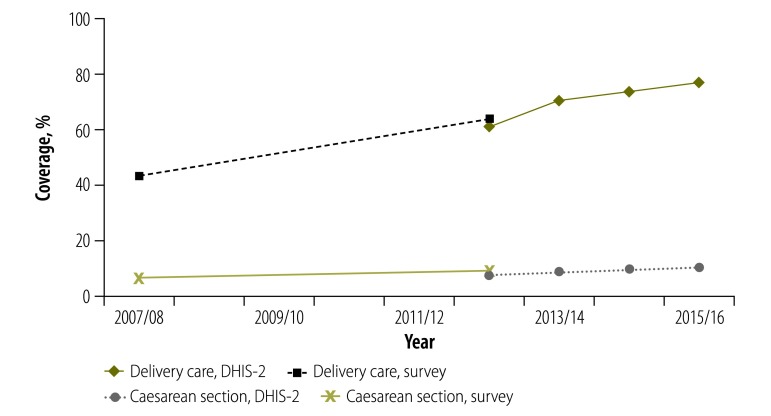
**Facility-based delivery coverage and caesarean section delivery rate comparing health facility and survey data, Kenya, 2007/08 to 2015/16**

Based on the adjusted facility data, first antenatal visit coverage was near-universal and close to the demographic and health survey results (Table 3 and Fig. 2). The consistency between facility and survey data was less satisfactory for the proportion of pregnant women who made four or more antenatal care visits. Household survey data (from two demographic and health surveys,^18^^,^^22^ and also from the Kenya malaria indicator survey 2015)^21^ showed an increase to 61.5% for the three years preceding the survey (midpoint shown in Fig. 3). The facility reporting data also showed an increase during the period 2012/13 to 2015/16 but at a lower level than the surveys. Since this is unlikely to be due to a problem with the size of the target population, it could be attributed to underreporting of four antenatal visits by health facilities or over-reporting of the number of visits in household surveys.

DHIS-2: district health information software, version 2.0.

Notes: Health-facility data were from routine reporting via DHIS 2 (district health information software, version 2.0) for fiscal years (1 July to 30 June) and aggregated for the 47 counties. Survey data were from the Kenya demographic and health survey 2014.^18^ Coverage denominators for facility data for 2013/14 and 2015/16 are in Table 3. Denominators for survey data for 2011/12 and 2009/10 are in Table 3 and for 2007/08 *n* = 3101 births.

DHIS-2: district health information software, version 2.0.

Notes: Health-facility data were from routine reporting via DHIS 2 (district health information software, version 2.0) for fiscal years (1 July to 30 June) and aggregated for the 47 counties. Survey data were from the Kenya demographic and health survey 2008‒09,^22^ Kenya demographic and health survey 2014^18^ and Kenya malaria indicator survey 2015.^21^ Coverage denominators for facility data for 2013/14 and 2015/16 are in Table 3. Denominators for survey data for 2011/12 and 2009/10 are in Table 3 and for 2007/08 *n* = 3101 births.

**Fig. 4:**
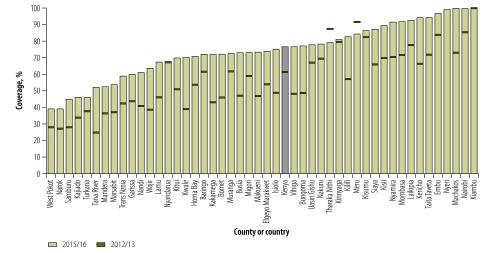
**Facility-based delivery coverage from health-facility data, by county, Kenya, 2012/13 and 2015/16**

Notes: Health-facility data were from routine reporting via DHIS 2 (district health information software, version 2.0) for fiscal years (1 July to 30 June). Adjusted values are shown, aggregated by county. Coverage denominators are in Table 5. Kenya, shown in grey value is the weighted mean of the 47 counties.

The facility data showed an increase in the proportion of women delivering in health facilities from 61.3% (815 959/1 325 124 deliveries) in 2012/13 to 76.9% (1 049 285/1 357 956 deliveries) in 2015/16, up from the survey estimate for 2012‒2014 of 64.0% (6 642/10 378 births) of births in health facilities. The variation in coverage of facility-based delivery by county was considerably greater than that for vaccination coverage, with values over 90% in ten counties and less than 60% in nine counties in 2015/16 (Fig. 4). Health facility delivery coverage was higher in 2015/16 than in 2012/13 in 44 of the 47 counties. The increase was large in almost all counties, and often greater in the lower coverage counties. 

**Table 5:** Numerators and denominators for calculating coverage of facility deliveries from health-facility data, by county, Kenya, 2012/13 and‒ 2015/16

County or country	Year 2012/13		Year 2015/16	
	No. of women giving birth	No. of health facility deliveries	No. of women giving birth	No. of health facility deliveries
Baringo	19 798	12 215	19 218	13 885
Bomet	23 998	11 012	24 292	17 632
Bungoma	61 472	29 943	57 503	44 620
Busia	31 364	14 825	27 104	19 875
Elgeyo Marakwet	15 239	8 171	14 395	10 683
Embu	13 166	11 058	13 260	12 897
Garissa	16 872	7 362	19 319	11 656
Homa Bay	38 036	20 394	36 193	25 751
Isiolo	6 133	3 008	6 542	4 926
Kajiado	30 223	10 237	33 185	15 402
Kakamega	63 612	27 239	59 571	43 152
Kericho	25 027	16 671	23 317	22 068
Kiambu	46 396	53 723	55 616	59 099
Kilifi	46 407	26 183	47 765	39 714
Kirinyaga	11 376	9 087	11 927	9 711
Kisii	38 434	26 875	36 663	32 920
Kisumu	36 911	30 542	35 019	30 329
Kitui	30 062	15 285	27 886	19 527
Kwale	29 736	11 633	29 784	20 942
Laikipia	13 878	10 820	14 763	13 712
Lamu	4 282	1 977	4 528	3 060
Machakos	29 214	21 306	29 448	29 628
Makueni	23 215	10 789	20 781	15 310
Mandera	21 242	7 656	26 551	14 023
Marsabit	12 837	4 759	13 385	7 220
Meru	35 466	32 607	36 470	30 905
Migori	42 170	24 792	42 704	31 404
Mombasa	29 939	21 458	33 748	31 079
Muranga	22 119	13 674	20 806	15 158
Nairobi	121 266	103 697	136 384	138 363
Nakuru	59 304	41 100	62 834	49 451
Nandi	23 339	9 567	21 297	13 136
Narok	38 516	10 474	42 251	16 795
Nyamira	19 441	13 705	20 472	18 814
Nyandarua	15 245	10 276	15 460	10 549
Nyeri	16 699	17 063	15 506	15 437
Samburu	10 595	2 978	10 440	4 728
Siaya	32 197	21 285	27 959	24 431
Taita Taveta	7 531	5 419	8 056	7 638
Tana River	8 675	2 144	9 839	5 159
Tharaka Nithi	9 854	8 632	9 368	7 445
Trans Nzoia	27 036	11 470	28 719	16 974
Turkana	28 345	10 743	34 267	15 936
Uasin Gishu	30 917	20 807	33 484	26 219
Vihiga	18 561	9 004	18 690	14 408
Wajir	17 743	6 872	19 853	12 686
West Pokot	27 861	7 811	28 122	11 157
Kenya^a^	1 331 750	815 959	1 364 745	1 049 285

^a^ Weighted county mean.

Notes: Health facility data are for fiscal years (1 July to 30 June).

The number of caesarean sections per 100 deliveries in the population also increased from 7.8 (103 785/1 325 124) in 2012/13 to 10.8 (147 463/1 357 956) in 2015/16 (Table 3), corresponding to the increased proportion of women delivering in health facilities. 

## Discussion

Health-facility data obtained from routine reporting systems are an important tool for assessing progress at subnational levels. This study presented a systematic approach to analysing routine health-facility data, focusing on data quality assessment and adjustment and obtaining denominators from data for interventions with near-universal coverage. Applying the method to Kenya showed that health-facility data can provide up-to-date information to monitor recent subnational and national coverage trends for key maternal and child health indicators. This study was conducted as part of the midterm review of the implementation of the Kenya health sector strategic and investment plan 2014–2018.^23^ In this plan the assessment of progress and performance towards the midterm targets in mid-2016 relied heavily on facility data because the last survey with maternal and child health indicators took place in 2014.

Our results provide important information on the maternal and child health component of the implementation of national and subnational health plans. Vaccination coverage rates stagnated or declined modestly, but were still at a high level. In most counties coverages were lower in 2015/16 than four years earlier. The Kenya strategic plan targets and the global goal of reaching and sustaining 90% national full vaccination coverage and 80% in every district or equivalent administrative unit for all vaccines included in the national programme^24^ were far from being met.

Deliveries in a health facility increased rapidly during the period 2012/13 to 2015/16. While household surveys showed a major increase to 64.0% before the Kenya strategic plan, the facility data indicated a continued national increase to 76.9% in 2015/16, driven by increases in 44 of the 47 counties. In 2013, Kenya introduced a free maternity initiative in all public health facilities, to encourage women to deliver in facilities. Even though it is not possible to wholly attribute the current trends to this initiative, the results obtained from the facility data are encouraging, confirming continued rapid increases in deliveries in health facilities. Further efforts are needed to concentrate in the nine counties with more than 40% of deliveries occurring at home (mostly located in the northern and more sparsely populated areas of Kenya) and in the counties with the largest numbers of home deliveries (located in western Kenya). Furthermore, caesarean section rates were increasing in almost all counties, proportional to the increases in institutional deliveries. Coverage of antenatal care with at least four visits was also increasing but much slower than delivery care coverage and was still only just over 50% in 2015/16 according to the facility data. Household survey data, based on recall by mothers, may overestimate the number of antenatal visits.

Analysis of the health-facility data was possible due to several factors present in Kenya that provide lessons for many other countries now implementing DHIS 2. This also highlights the limitations of this type of analysis. First, the health ministry, both at national and county levels, has a strong commitment to the health facility reporting system. The government made it mandatory that all programmes use the same system for collection of facility-based indicators to ensure that the systems are interoperable. The only exception to date is the disease surveillance system which is not yet fully integrated. The health ministry is also strongly committed to sharing the DHIS 2 data, in line with the Kenya government’s open data initiative.^25^ The devolution has stimulated the interest in county-level monitoring.

Second, the reporting system has been functioning well. Reporting rates are high and have increased to over 90%. The private sector is included, even though reporting rates are still lower than for the public sector. We adjusted the numbers of reported events for incomplete reporting by making assumptions about the extent to which non-reporting facilities would be different from reporting facilities and using the survey data as an external validity check. This is a somewhat arbitrary process, but the impact of the adjustments is generally relatively modest if reporting rates are high. If reporting completeness is below 80%, adjustment procedures will have a greater impact and facility statistics will become less reliable. Other methods to adjust for missing values include geospatial methods, which have for instance been used in Kenya for estimating outpatient visits rates from facility data.^26^

Third, the facility data were of good quality, as shown by good consistency over time, consistency across indicators and external comparison with surveys. In Kenya, the districts or counties, supported by the health ministry, usually compute the reporting rates, check for data inconsistencies and do follow-ups to ensure high levels of reporting and accuracy of data. DHIS 2 now includes a standardized module to check for inconsistencies and outliers which makes it easier for staff at county and national health offices to identify problems and follow up with action.^15^ Previous research also indicated relatively good quality of facility data in Kenya.^27^

A fourth factor was the availability of an accurate estimate of the target population for the indicator, or the denominator of the coverage estimate. In Kenya, many counties had identified major problems with the denominators provided as part of the official population projections based on the 2009 census. Here, we used county reports on the number of vaccinations with first dose of pentavalent vaccination to obtain denominators for the maternal and child health indicators. This can only be done if the numerators are accurate, with high reporting rates and good quality of data. Supplemental immunization activities, in which children are vaccinated outside of clinical settings,^28^ are not likely to affect the usefulness of first dose of pentavalent vaccination numbers to obtain a denominator.

Fifth, recent (up to 3‒4 years ago) household survey data are necessary to be able to calibrate the denominators. It has to be kept in mind, however, that surveys are not the absolute gold standard, as the survey results are affected by sampling error (which can be large, especially at subnational levels) and non-sampling error related to recall bias or the quality of the survey implementation.

Lastly, a specific advantage for this study was that Kenya’s unit of analysis – the county – is relatively large (almost all counties have populations exceeding 500 000) which helps to obtain more stable estimates of numerators and denominators. The methods, however, have potential for use in smaller populations, such as subcounties or districts, as target populations are based on the actual volume of health services provided to the same population rather than population projections.

Surveys will continue to be necessary to provide population-based data on a range of maternal and child health coverage indicators and determinants. However, the introduction of national web-based information systems for health-facility data provides an opportunity for more frequent monitoring of progress at the national and subnational levels. This study shows how improvements in the timeliness, completeness and accuracy of a new web-based reporting system can provide a sound basis for subnational and national statistics on key maternal and child health indicators. This approach can be extended to obtain statistics for other indicators, such as stillbirth rates, postnatal care coverage and outpatient attendance.^29^ The main application of this approach lies at subnational levels where regular monitoring of progress and performance has the greatest potential to improve service delivery and targeting of interventions.
